# Simulating the effect of climate change on soil microbial community in an *Abies georgei* var. smithii forest

**DOI:** 10.3389/fmicb.2023.1189859

**Published:** 2023-06-02

**Authors:** Fangwei Fu, Jiangrong Li, Yueyao Li, Wensheng Chen, Huihui Ding, Siying Xiao

**Affiliations:** ^1^Research Institute of Tibet Plateau Ecology, Tibet Agriculture and Animal Husbandry University, Nyingchi, Tibet, China; ^2^Key Laboratory of Forest Ecology in Tibet Plateau, Ministry of Education, Nyingchi, Tibet, China; ^3^National Key Station of Field Scientific Observation and Experiment, Nyingchi, Tibet, China; ^4^Key Laboratory of Alpine Vegetation Ecological Security in Tibet, Nyingchi, Tibet, China; ^5^State Key Laboratory of Tibetan Plateau Earth System, Resources and Environment (TPESRE), Institute of Tibetan Plateau Research, Chinese Academy of Sciences, Beijing, China

**Keywords:** soil transplantation, soil microorganisms, Sygera Mountains, climate change, covariance network

## Abstract

Qinghai–Tibet Plateau is considered a region vulnerable to the effects of climate change. Studying the effects of climate change on the structure and function of soil microbial communities will provide insight into the carbon cycle under climate change. However, to date, changes in the successional dynamics and stability of microbial communities under the combined effects of climate change (warming or cooling) remain unknown, which limits our ability to predict the consequences of future climate change. In this study, *in situ* soil columns of an *Abies georgei* var. *smithii* forest at 4,300 and 3,500 m elevation in the Sygera Mountains were incubated in pairs for 1 year using the PVC tube method to simulate climate warming and cooling, corresponding to a temperature change of ±4.7°C. Illumina HiSeq sequencing was applied to study alterations in soil bacterial and fungal communities of different soil layers. Results showed that warming did not significantly affect the fungal and bacterial diversity of the 0–10 cm soil layer, but the fungal and bacterial diversity of the 20–30 cm soil layer increased significantly after warming. Warming changed the structure of fungal and bacterial communities in all soil layers (0–10 cm, 10–20 cm, and 20–30 cm), and the effect increased with the increase of soil layers. Cooling had almost no significant effect on fungal and bacterial diversity in all soil layers. Cooling changed the structure of fungal communities in all soil layers, but it showed no significant effect on the structure of bacterial communities in all soil layers because fungi are more adapted than bacteria to environments with high soil water content (SWC) and low temperatures. Redundancy analysis (RDA) and hierarchical analysis showed that changes in soil bacterial community structure were primarily related to soil physical and chemical properties, whereas changes in soil fungal community structure primarily affected SWC and soil temperature (Soil Temp). The specialization ratio of fungi and bacteria increased with soil depth, and fungi were significantly higher than bacteria, indicating that climate change has a greater impact on microorganisms in deeper soil layers, and fungi are more sensitive to climate change. Furthermore, a warmer climate could create more ecological niches for microbial species to coexist and increase the strength of microbial interactions, whereas a cooler climate could have the opposite effect. However, we found differences in the intensity of microbial interactions in response to climate change in different soil layers. This study provides new insights to understand and predict future effects of climate change on soil microbes in alpine forest ecosystems.

## 1. Introduction

Soil microbes play an important role in ecosystem material cycling, energy flow, and organic matter conversion (Nottingham et al., 2019; Yu et al., 2019), and their response to climate change may profoundly affect soil carbon storage (Chen et al., 2015; Nottingham et al., 2019; Zheng et al., 2020). A comprehensive understanding of changes in soil microbial community structure would provide insight into the carbon cycle under climate change (Zheng et al., 2020). Climate change includes simultaneous changes in temperature and precipitation, which may affect the composition of soil microbial communities. However, few cases have been found on the previously reported two-factor experiments combining temperature and precipitation changes in warming experiments, and their results vary greatly in different ecosystems (Hu et al., 2020; Rasmussen et al., 2020; Simon et al., 2020). At this stage, field warming experiments are mostly recorded using open-top box observations, which inevitably change the microclimate within the box, while the use of anthropogenic periodic watering methods cannot simulate naturally occurring precipitation events throughout the year (Ma et al., 2023). These uncertainties limit our comprehensive understanding of the mechanism by which soil microbes on the Tibetan Plateau will respond to future climate change.

Soil microbes are closely related to physicochemical factors such as soil organic matter (SOM), but soil microbes are likely to be highly variable at depth because SOM and other physicochemical factors are highly variable at depth (Song et al., 2022). However, studies related to the effects of warming on SOM decomposition have focused on topsoil (Cheng et al., 2017; Song et al., 2022), but most SOM is old and stored in subsoil. Previous studies have shown that the response of soil physicochemical processes to climate change is profoundly influenced by soil depth (Liu et al., 2022; Song et al., 2022). Therefore, changes in the composition of soil microbial communities, which are closely related to soil microclimate with regard to the effectiveness of soil substrates, may also be related to soil depth (Canarini et al., 2016). However, whether and to what extent soil depth affects the response of soil microbial communities to climate change has not been well documented (Cheng et al., 2017).

Soil transplantation experiments can expose microbial communities to natural climatic conditions and provide an effective experimental platform to study microbial responses to climate change (Lazzaro et al., 2011; Djukic et al., 2013). Moreover, the reciprocal reset experiment of exchanging whole soil plants along an elevation gradient provides relatively natural gradient warming and cooling without a high energy supply (Egli et al., 2004; Ishizuka and Goto, 2011). Therefore, the reciprocal displacement experiment is a rigorous method to demonstrate the effects of climate change because it allows the simultaneous application of warming and cooling as well as minimizes chemical and mechanical disturbances (Egli et al., 2004). In general, the temperature can increase, and precipitation can decrease in transplants to lower elevations (warming climate). By contrast, temperature can decrease, and precipitation can increase in transplants to higher elevations (cooling climate), with soils transplanted to higher/lower elevations having a cooler/warmer climate profile (Rui et al., 2015). Thus, the effect of climate on microbial communities can be analyzed. The response of microbial communities to climate change simulated using soil transplantation has been reported (Puissant et al., 2015; Nottingham et al., 2021; Song et al., 2022), but the mechanism of action of soil microbes in response to climate change (cooling or warming) remains unclear.

Previous studies (Vanhala et al., 2011) have shown that differences in soil microbial community structure and community function were found after transplanting forest soils from northern China to corresponding forest stations on the warmer southern border for 2 years. Liu et al. (2015) found changes in soil nitrogen composition, microbial biomass, and community structure after transplanting from southern (subtropical climate zone) to central (warm-temperate climate zone) and northern (cool-temperate climate zone) China for 4 years. Zumsteg et al. (2013) found that by transferring fine granitic sediments and sampling them regularly, microbial activity responded only to soil transfer from warm and dry sites to colder and wetter sites, whereas bacterial and fungal community structures responded to transfer in both directions. These studies have shown that soil transplantation alters soil nutrient, soil microbial biomass, functional diversity, community structure, and composition (Vanhala et al., 2011; Zumsteg et al., 2013; Zhao et al., 2014; Liu et al., 2015), revealing that climate change has significant effects on soil microbial communities. However, these studies usually focus on the role of univariate diversity or composition indicators, ignoring that ecosystem processes are carried out via complex webs of co-occurrence networks among microbiome members (Chen et al., 2022). Previous studies have demonstrated that microbial communities are structured, and they form complex interconnected networks through competition, facilitation, and inhibition (Barberán et al., 2012; de Vries et al., 2018). These networks are influenced by direct and indirect interactions among microbial species, which are more important than environmental factors in shaping community structure (Gokul et al., 2016). Microbial network analysis can provide a comprehensive understanding of these complex community structures and species interactions, even in distantly related communities, and can reveal the mechanisms by which they are influenced by environmental factors (Fuhrman, 2009; Barberán et al., 2012). However, the relationship among climate change, network complexity, and stability is a challenging topic in theoretical ecology, which has not been fully studied in the field of microbial ecology, and the response of microbial ecological networks to future climate change is poorly understood (Toju et al., 2017; García-Palacios et al., 2018).

The Qinghai–Tibet Plateau accumulates 3.84 × 10^9^ tons of organic carbon, accounting for about 21% of China’s soil carbon stocks (Zhao et al., 2018). In addition, the southeastern region of the Qinghai–Tibet Plateau is characterized by a vast expanse of dark coniferous forests, primarily dominated by Spruce species (Miehe et al., 2007; Liang et al., 2011). This region is a typical example of a plateau mountainous primary forest area, exhibiting a rich abundance of forest resources and a distinct vertical zonation. Forests play a crucial role in soil and water conservation, water shading, and maintaining regional ecological balance. Furthermore, the region is highly sensitive to climate change (Castro et al., 2010; Liang et al., 2010; Luo et al., 2014). Therefore, studying the impact of climate change on the structure of soil microbial communities will help to understand the carbon cycle of alpine mountain forest systems under climate change conditions. The use of elevation gradients in the alpine ecosystems of the region as simulations of climate gradients can provide a natural model for analyzing the impact of climate change on ecosystem structure and function, and this approach has been successfully used to document the effects of global change on vegetation (Palareti et al., 2016; Yuan et al., 2021) and soil biogeochemical processes (Nottingham et al., 2021). This study aimed to elucidate the response of soil bacterial and fungal communities to climate warming and cooling in the forest ecosystem of *Abies georgei* var. *smithii* in the Sygera Mountains of southeastern Tibet, China. We hypothesized (1) that soil transplantation would alter the diversity (Shannon index) and community structure of bacteria and fungi, with soil temperature and soil moisture content being the primary drivers of this change. Soil fungi have a unique mycelial structure, and they are more resistant to low-temperature stress than bacteria; thus, experimental climate cooling affected soil bacteria more than fungi and vice versa; (2) considering that a high temperature stimulates various biological interactions (predation, parasitism, competition, and symbiosis), experimental warming will increase the complexity of species associations, leading to complex ecological networks, whereas cooling will have the opposite effect on soil microbial communities (symmetric response).

## 2. Materials and methods

### 2.1. Study area

The Sygera Mountains in southeastern Tibet are located in Nyingchi County, southeastern Tibet (29°5′-29°57′ N, 94°25′-94°45′ E), in the northwestern part of the great bend of the Yarlung Tsangpo River, which belong to the remnant of the Tanggula Range. The climate of the region belongs to the typical subalpine temperate semi-humid climate, with distinct dry and wet seasons. The average annual temperature is −0.73°C, and the average annual precipitation is 1,134 mm, primarily concentrated in June–September. The main vegetation types include alpine sparse mat vegetation, dark coniferous forest dominated by *Picea jezoensis–A. nephrolepis*, evergreen broadleaf forest dominated by *Quercus aquifolioides*, and deciduous broadleaf forest dominated by poplar birch forest (Luo et al., 2014). The studying stand was a mature primary forest with *A. georgei* var. *smithii* as a single tree layer, with an average height of 27.68 m, an average diameter at breast height of 42.59 cm, and a denseness of 0.4–0.9 in the main stand. The understory is undulating, and the soil is acidic brown loam with a well-developed moss layer. At elevation of 3,500 m, *Abies georgei* var. *smithii* is densely distributed, forming a pure forest, whereas elevation of 4,300 m is the upper limit of the pure forest distribution of *Abies georgei* var. *smithii*.

At an elevation of 3,500 m, the average annual temperature is 4.9°C, with an average annual precipitation of 648.9 mm. The warmest month (July) has an average temperature of approximately 13.70°C, whereas the coldest month (January) has an average temperature of approximately −1.91°C, resulting in an annual temperature difference of 15.60°C. The understory of this area is dominated by several plant species, including *Rhododendron aganniphum* var. *schizopeplum*, *Lonicera inconspicuous*, *R. uvariifolium*, *R. hirtipes*, *Ribes glaciale*, *Polygonatum cirrhifolium*, *Natum cirrhifolium*, *Streptopus simplex*, *Hemiphragma heterophyllum*, and *Polygonum filicaule*.

At an elevation of 4,300 m, the average annual temperature is 1.23°C, with an average annual precipitation of 999.2 mm. The warmest month (July) has an average temperature of approximately 8.53°C, whereas the coldest month (January) has an average temperature of approximately −6.48°C, resulting in an annual temperature difference of 15.01°C. The forest understory in this area is primarily composed of several plant species, including *Potentilla fruticosa*, *Rubus*, *R. nyingchiense*, *Cassiope selaginoides*, *Cremanthodium reniforme*, and *Rhodiola fastigiata.*

### 2.2. Sample plot setup and soil sample collection

On June 22, 2020, three standard sample plots with a size of 30 m × 30 m were set up at 3500 and 4,300 m above sea level, with each sample plot spaced at least 100 m. After setting up the sample plots, each sample plot was evenly divided into a grid size of 5 m × 5 m. After establishing the sample plots, each sample plot was evenly and equally divided into a grid of 5 m × 5 m, and then a PVC pipe (20 cm in diameter and 45 cm in length) was driven vertically into the soil in the middle of each grid until the distance between the mouth of the PVC pipe and the horizontal ground was 10 cm. Six PVC pipes were set up in each sample plot, and then three PVC pipes full of soil samples were carefully removed from each sample plot (to avoid leakage of soil samples from the bottom of the pipes). Afterward, the bottom of the PVC pipes was sealed with plastic sheeting. Then, the soil at 3500 m above sea level and 4,300 m above sea level was matched and buried underground with the same PVC pipe opening at a distance of 10 cm from the horizontal ground to simulate colder and warmer climates. The remaining three PVC pipes in each sample plot were then used as a control group. After 1 year of incubation, soil samples were collected from three different soil layers (0–10, 10–20, and 20–30 cm) on June 22, 2021, using soil *in situ* augers with an internal diameter of 5 cm, and a total of 108 soil samples were collected (a total of 36 PVC pipes; one soil sample was drilled from each PVC pipe in the 0–10 cm, 10–20 cm, and 20–30 cm soil layers). In addition, a total of three replicates were collected. The collected soil was passed through a 2-mm sieve to remove plant roots and stones. The remaining soil was then divided into two portions. One portion was transferred to a sterile bag and kept in an ice box for transportation to the laboratory. Upon arrival, it was frozen at −80°C for further analysis. These samples were immediately sent to the institution on the second or third day for soil microbial DNA extraction. The other part was used to determine the physicochemical properties of the soil.

### 2.3. Determination of soil physical and chemical properties

Soil pH was determined using the potentiometric method with a water–soil ratio of 2.5:1. Soil available phosphorus (AP) was analyzed using the molybdenum antimony anti-colorimetric method with HCl-NH4F leaching and spectrophotometry. Total phosphorus (TP) and AP were analyzed using the molybdenum antimony anti-spectrophotometric method (Song et al., 2022). Soil total organic carbon (TOC) was determined using a TOC analyzer. Soil ammonium nitrogen (NH_4_^+^-N) and nitrate nitrogen (NO_3_^−^-N) were determined by leaching with 1 mol/L of KCl at a soil–water ratio of 1:5 by using a continuous flow analyzer. Total nitrogen (TN) was determined using a carbon and nitrogen elemental analyzer, whereas soil available nitrogen (AN) was determined using alkaline hydrolyzable nitrogen. The soil water content (SWC) and temperature (Temp) were measured using a soil moisture sensor and a soil temperature sensor, respectively. Meteorological data from January to December 2021 were collected at a depth of 0–30 cm and recorded once every 10 min using a HOBO H21-USB data collector manufactured by the Onset Company, United States. Meteorological data were downloaded every other month. An overview of the meteorological instruments used in this study is provided in Table 1.

**Table 1:** Overview of meteorological monitoring instruments.

Item	Sensor model	Range	Accuracy	Resolutio*n*	Manufacturer
Soil temperature	HOBO S–TMB–M006	–40–100°C	±0.20°C	0.03°C	USA Onset
Soil moisture	HOBO S–SMD–M005	0–570 m^3^/m3	±3.30%	0.08%	USA Onset

### 2.4. DNA extraction, amplicon sequencing, and bioinformatic analyses

Soil DNA extraction, PCR amplification, and Illumina sequencing were outsourced to Shanghai Meiji Biomedical Technology Co., Ltd. (Shanghai, China). Total DNA was extracted from soil samples using the QJ-Soil kit. The concentration and purity of the extracted DNA were determined using NanoDrop 2000 and 1% agarose gel electrophoresis. The V3–V4 region of the bacterial 16SrRNA gene was amplified using the primers 338F (5′-ACTCCTACGGGAGGCAGCAG-3′) and 806R (5′-GGACTACHVGGGTWTCTAAT-3′). The fungal internal transcribed spacer (ITS) was amplified using the primers ITS1F (5′-CTTGGTCATTTAGAGTAA-3′) and ITS2R (5′-GCTGCGTTTCTTCATCGATGC-3′). The PCR amplification of the 16S rRNA gene was performed as follows: initial denaturation at 95°C for 3 min, followed by 27 cycles of denaturation at 95°C for 30 s, annealing at 55°C for 30 s, extension at 72°C for 45 s, single extension at 72°C for 10 min, and end at 4°C. The PCR mixture contains 4 μL of 5× TransStart FastPfu buffer, 2 μL of 2.5 mM dNTPs, 0.8 μL of forward primer (5 μM), 0.8 μL of reverse primer (5 μM), 0.4 μL of TransStart FastPfu DNA polymerase, 10 ng of template DNA, and up to 20 μL of ddH_2_O. PCR reactions were performed in triplicate. The PCR product was extracted from 2% agarose gel and purified using the AxyPrep DNA Gel Extraction Kit (Axygen Biosciences, Union City, CA, United States) according to the manufacturer’s instructions and quantified using the Quantus™ Fluorometer (Promega, United States). Purified amplicons were pooled in equimolar and paired-end sequenced on an Illumina MiSeq PE300 platform platform (Illumina, San Diego, United States) in accordance with the standard protocols by Majorbio Bio-Pharm Technology Co. Ltd. (Shanghai, China).

Raw 16S rRNA gene sequencing reads were demultiplexed, quality filtered by fastp version 0.20.0 (Chen et al., 2018), and merged by FLASH version 1.2.7 (Salzberg, 2011) in accordance with the following criteria: (i) 436 bp reads were truncated at any site receiving an average quality score of <20 over a 50 bp sliding window, and truncated reads shorter than 50 bp and reads containing ambiguous characters were discarded; (ii) only overlapping sequences longer than 10 bp were assembled in accordance with their overlapped sequence. The maximum mismatch ratio of the overlap region is 0.2. Reads that could not be assembled were discarded; (iii) samples were distinguished in accordance with the barcode and primers, and the sequence direction was adjusted. Barcode matching was also performed, and two nucleotides mismatch during primer matching. Operational taxonomic units (OTUs) with 97% similarity cutoff (Stackebrandt and Goebel, 1994; Wang et al., 2007) were clustered using UPARSE version 7.1 (Stackebrandt and Goebel, 1994), and chimeric sequences were identified and removed.

### 2.5. Operational taxonomic unit clustering and notes

In this study, a total of 6,766,956 high-quality and chimera-free reads with an average length of 412 bp were obtained by pyrosequencing across the 108 soil samples. To reduce the amount of redundant calculation in the process of analysis, we counted the repetitive sequence and extracted the non-repetitive sequence. We used Vsearch software to remove the once-detected sequences (Singleton sequences). The sequences with more than 97% similarity were gathered into one operational taxonomic unit (OTU), the chimeric sequence was removed, and the OTU representative sequence was used for subsequent species annotation. We used the Ribosomal Database Project (RDP) and SLIVA databases to classify and identify bacteria and fungi, respectively. The raw sequencing reads are available at the Sequence Read Archive (SRA) database of the National Center for Biotechnology Information (NCBI) under accession numbers SUB13069704: PRJNA955721.

### 2.6. Data statistics

One-way ANOVA was used to test the differences in soil physical and chemical properties before and after climate warming and climate cooling, and the significance was determined at the 0.05 level using the least significant difference test. The Shapiro–Wilk test and Levene test were used to evaluate the normality and chi-square of data. The alpha diversity index (Shannon index) was calculated on the basis of the OTU information. The larger the Shannon index, the higher the community diversity. The Chao1 index was used to estimate species richness, which reflects the richness of the sample, and the higher its value, the higher the richness of the community (Liu et al., 2018). Histograms were plotted at the phylum level to show the relative abundance of each microorganism, and color changes were used to reflect the similarities and differences in the community composition of different samples at the phylum level. The bacterial and fungal diversity in soil was analyzed using non-metric multidimensional scaling (NMDS) (Wang et al., 2022) based on the Bray–Curtis distance algorithm, and then analysis of similarity (ANOSIM) (Li et al., 2019) was used to determine the differences in bacterial and fungal community compositions in soil, which can determine the differences in bacterial and fungal communities in soil before and after climate warming. The relationship between soil physicochemical properties and soil bacterial and fungal communities was investigated by redundancy analysis (RDA) (Ji et al., 2021). The importance of environmental variables was evaluated using the “rdacca.hp” package in R using a hierarchical partitioning approach (Zhang et al., 2022). The relative importance of soil factors on microbial Shannon diversity was evaluated using the random forest (RF) in “rfPermute” package (Ji et al., 2021) based on the increase of mean square error and *p*-values. Network analysis (Zhong et al., 2022) was used to study the biological interactions among microbial populations, showing the mechanism by which OTUs in soil microbial communities interact with one another before and after climate warming and cooling. The inner relationship (networks among taxa within each microhabitat type) was based on network co-occurrence analysis of OTUs with a relative abundance of >0.001. Each dot represents a bacterial or fungal phylotype, and the links indicate statistical significance (*p* < 0.01). Spearman correlation coefficient matrices were calculated at the genus level using the “Hmisc” (Zhong et al., 2022) software package in R. Each point represents an independent sample, and connections represent statistical significance (*p* < 0.01). Spearman correlation coefficients had a correlation coefficient of >0.6. In addition, the number of nodes and edges, average path length, network diameter, cumulative degree distribution, clustering coefficient, and modularity were calculated in accordance with a previous study using the function “network()” in the R package “ggClusterNet” (Yuan et al., 2018). The network was visualized using Gephi (0.9.2). All statistical analyses were performed in R4.1.2.

## 3. Results

### 3.1. Effect of soil transplantation on soil physicochemical properties

Transplanting soil from a high elevation (4,300 m above sea level) to a low elevation (3,500 m above sea level) did not result in significant changes in ammonia nitrogen (NH_4_^+^-N), nitrate nitrogen (NO_3_^−^-N), AP, pH, carbon-to-nitrogen ratio (C/N), TN, and TOC (*p* > 0.05, Table 2). However, significant decreases in TP, AN, and SWC, as well as a significant increase in soil temperature (Soil Temp; *p* < 0.05, Table 2), were observed in the 0–10 cm soil layer (the mean values for the control group at 4300 m were 0.63%, 0.61 g/kg, 31%, and 4.4°C, and those for the experimental group at M4300-3500 m were 0.51%, 0.49 g/kg, 24%, and 9.06°C, respectively). In the 10–20 cm soil layer (the mean values for the control group at 4300 m were 4.31 mg/kg, 0.32 g/kg, 21.51, 48%, and 4.36°C, and those for the experimental group at M4300-3500 m were 2.49 mg/kg, 0.15 g/kg, 18.62, 32%, 8.91°C, respectively), significant reductions in AP, AN, C/N, TOC, TP, and SWC, as well as a significant increase in Soil Temp, were observed (*p* < 0.05, Table 2). In the 20–30 cm soil layer, (the mean values for the control group at 4300 m were 2.15 mg/kg, 5.32°C, 49%, 5.09 mg/kg, and 22.78, and those for the experimental group at M4300-3500 m were 10.46 mg/kg, 8.91°C, 24%, 2.43 mg/kg, and 19.41, respectively), significant increases in NH_4_^+^-N and Soil Temp, as well as significant decreases in SWC, AP, and C/N, were observed (*p* < 0.05, Table 2).

**Table 2:** Effect of soil transplantation on soil physicochemical properties.

Soil Depth(cm)	Indicator	Elevation (m)			
		3,500	M3500–4300	4,300	M4300–3500
0–10 cm	NH4^+^–N(mg/kg)	23.03 ± 3.60^aA^	25.44 ± 2.86^aA^	26.65 ± 2.53^aA^	29.63 ± 2.65^aA^
	NO3^−^–N(mg/kg)	6.16 ± 1.93^bA^	12.16 ± 2.37^aA^	5.03 ± 1.46^aA^	4.95 ± 0.87^aA^
	TN(%)	0.49 ± 0.33^aA^	0.46 ± 0.31^aA^	0.47 ± 0.38^aA^	0.54 ± 0.68^aA^
	TOC(%)	10.20 ± 1.04^aA^	9.22 ± 0.61^aA^	9.65 ± 0.77^aA^	10.62 ± 1.86^aA^
	TP(g/kg)	0.44 ± 0.020^bB^	0.63 ± 0.038^aAB^	0.63 ± 0.030^aAB^	0.51 ± 0.038^bA^
	AP(mg/kg)	9.62 ± 1.87^aA^	7.14 ± 1.25^aA^	9.58 ± 1.79^aA^	8.61 ± 2.80^aA^
	AN(g/kg)	0.37 ± 0.04^aA^	0.49 ± 0.07^aA^	0.61 ± 0.06^aA^	0.49 ± 0.08^bA^
	PH	5.73 ± 0.13^aB^	5.56 ± 0.09^aB^	5.93 ± 0.11^aB^	5.60 ± 0.11^aB^
	C/N	20.57 ± 1.05^aA^	20.01 ± 0.34^aB^	20.63 ± 0.44^aB^	19.13 ± 0.96^bA^
	SWC(%)	24% ± 0.01^bB^	31% ± 0.01^aC^	31% ± 0.01^aC^	24% ± 0.01^bB^
	Temp(°C)	9.06 ± 0.81^aA^	4.4 ± 0.87^bC^	4.4 ± 0.87^bC^	9.06 ± 0.81^aA^
10–20 cm	NH4^+^–N(mg/kg)	13.07 ± 2.11^aB^	9.97 ± 2.18^bB^	13.35 ± 2.41^aB^	17.88 ± 1.42^bB^
	NO3^−^–N(mg/kg)	3.84 ± 0.76^aA^	4.96 ± 1.52^aB^	2.52 ± 0.67^aAB^	2.42 ± 0.61^aB^
	TN(%)	0.29 ± 0.02^aB^	0.32 ± 0.02^aB^	0.33 ± 0.02^aB^	0.26 ± 0.01^aB^
	TOC(%)	5.47 ± 0.59^bB^	6.85 ± 0.37^aB^	7.00 ± 0.47^aB^	4.79 ± 0.33^bB^
	TP(g/kg)	0.35 ± 0.01^bC^	0.58 ± 0.03^aB^	0.55 ± 0.02^aB^	0.30 ± 0.03^bB^
	AP(mg/kg)	3.29 ± 0.47^aB^	4.22 ± 0.69^aA^	4.31 ± 0.56^aB^	2.49 ± 0.18^bB^
	AN(g/kg)	0.13 ± 0.03^aB^	0.16 ± 0.0.03^aB^	0.32 ± 0.04^aB^	0.15 ± 0.01^bB^
	PH	6.18 ± 0.07^aA^	5.92 ± 0.89^aA^	6.02 ± 0.81^aAB^	5.96 ± 0.11^aA^
	C/N	19.20 ± 0.65^bA^	21.16 ± 0.47^aAB^	21.51 ± 0.32^aAB^	18.62 ± 0.540^bA^
	SWC(%)	32% ± 0.02^bA^	48% ± 0.01^aB^	48% ± 0.01^aB^	32% ± 0.02^bA^
	Temp(°C)	8.91 ± 0.68^aB^	4.36 ± 0.87^bB^	4.36 ± 0.87^bB^	8.91 ± 0.68^aB^
20–30 cm	NH4^+^–N(mg/kg)	6.75 ± 0.97^aB^	5.76 ± 2.70^aB^	2.15 ± 0.30^bC^	10.46 ± 1.99^aC^
	NO3^−^–N(mg/kg)	2.18 ± 0.49^aB^	3.02 ± 1.00^aB^	1.25 ± 0.41^aB^	1.28 ± 0.27^aB^
	TN(%)	0.19 ± 0.01^bC^	0.25 ± 0.01^aC^	0.20 ± 0.01^aC^	0.21 ± 0.01^aB^
	TOC(%)	4.11 ± 0.27^bC^	5.48 ± 0.26^aC^	4.55 ± 0.30^aC^	4.22 ± 0.20^aB^
	TP(g/kg)	9.62 ± 1.87^aA^	7.14 ± 1.25^aA^	9.58 ± 1.79^aA^	8.61 ± 2.80^aA^
	AP(mg/kg)	3.38 ± 0.37^aB^	5.05 ± 1.17^aA^	5.09 ± 0.56^aB^	2.43 ± 0.35^bB^
	AN(g/kg)	0.03 ± 0.006^bC^	0.10 ± 0.02^aB^	0.09 ± 0.01^aC^	0.100 ± 0.01^aB^
	PH	6.31 ± 0.07^aA^	6.00 ± 0.07^bA^	6.25 ± 0.08^aA^	6.14 ± 0.08^aA^
	C/N	20.85 ± 0.44^aA^	21.88 ± 0.49^aA^	22.78 ± 0.82^aA^	19.41 ± 0.42^bA^
	SWC(%)	24% ± 0.01^bB^	49% ± 0.01^aA^	49% ± 0.01^aA^	24% ± 0.01^bB^
	Soil Temp(°C)	8.91 ± 0.49^aB^	5.32 ± 0.91^bA^	5.32 ± 0.91^bA^	8.91 ± 0.49^aB^

Values are presented as mean ± standard deviation (*n* = 9). Based on one–way analysis of variance (ANOVA), means followed by the same letter for a given factor were not significantly different (*p* < 0.05 or *p* < 0.01). NH_4_^+^–N: ammonia nitrogen; NO_3_^−^–N: nitrate nitrogen; AP: available phosphorus; AN: available nitrogen; pH: soil pH; TOC: total organic carbon; TN: total nitrogen; TP: total phosphorus; SWC: soil water content; Soil Temp: soil temperature. Different lowercase letters indicate significant differences between different elevations for the same soil depth and different capital letters indicate significant differences between different subgroups for different soil depths for the same elevation (*p* < 0.05). M3500–4300, transplanting soil from elevation 3,500 to elevation 4,300 m; M3500–4300, transplanting soil from elevation 4,300 to elevation 3,500 m. M3500–4300, transplanting soil from elevation 3,500 to elevation 4,300 m; M3500–4300, transplanting soil from elevation 4,300 to elevation 3,500 m.

Transplanting soil from a low elevation (3,500 m above sea level) to a high elevation (4,300 m above sea level) resulted in a significant increase in nitrate nitrogen (NO_3_^−^-N), TP, and soil moisture content (SWC) in the 0–10 cm soil layer (the mean values for the control group at 3500 m were 6.16 mg/kg, 0.44, and 24%, and those for the experimental group at M3500-4300 m were 12.16 mg/kg, 0.63, and 31%, respectively; *p* < 0.05, Table 2). By contrast, all other soil physicochemical properties did not change significantly (*p* > 0.05, Table 2). In the 10–20 cm soil layer (the mean values for the control group at 3500 m were 13.07 mg/kg, 8.91°C, 5.47, 0.35, 32%, and 19.20, and those for the experimental group at M3500-4300 m were 9.97 mg/kg, 4.36°C, 6.85, 0.58, 48%, and 21.16, respectively), significant decreases in ammonia nitrogen (NH_4_^+^-N) and Soil Temp, as well as significant increases in TOC, TP, SWC, and C/N, were observed (*p* > 0.05, Table 2). In the 20–30 cm soil layer (the mean values for the control group at 3500 m were 0.19 g/kg, 4.11, 24%, 0.03 mg/kg, 6.31, and 8.91°C, and those for the experimental group at M3500-4300 m were 0.25 g/kg, 5.48, 49%, 0.1 mg/kg, 6.00, and 5.32°C, respectively), significant increases in TN, TOC, SWC, and AN (*p* < 0.05), as well as significant decreases in pH and Soil Temp, were observed (*p* > 0.05, Table 2).

### 3.2. Effect of soil transplantation on soil microbial diversity

**Figure 1:**
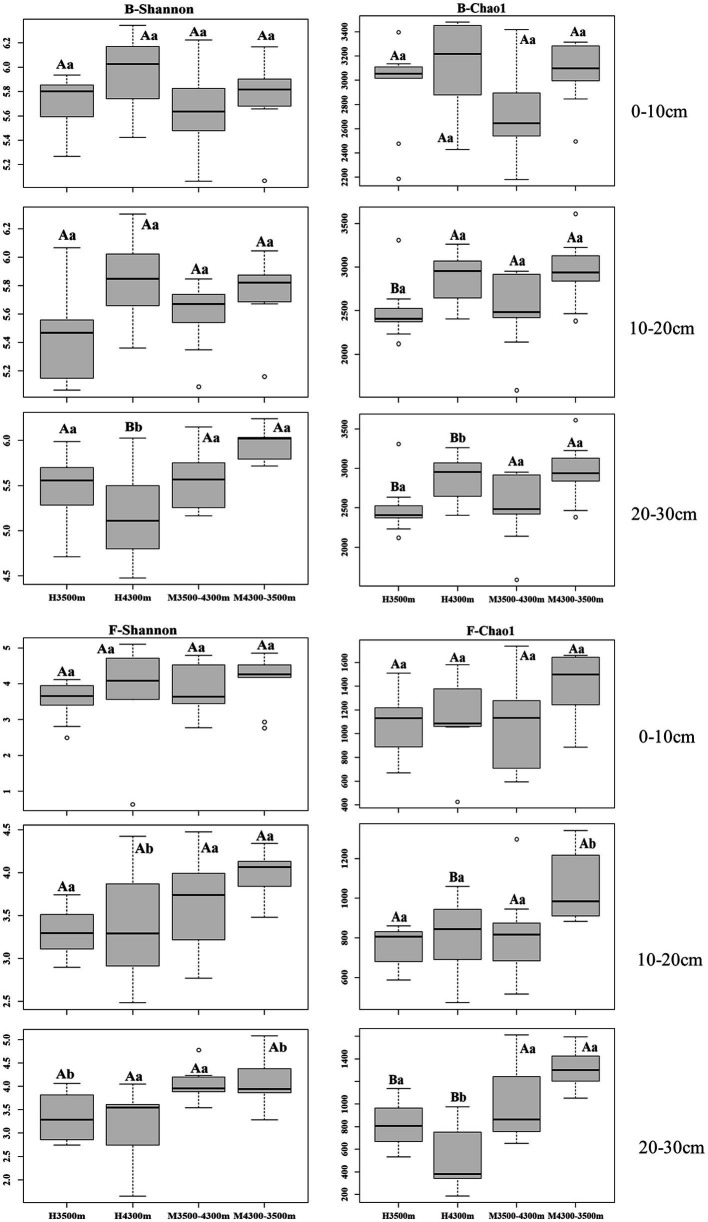
Alpha diversity of soil fungi and bacteria in different subgroups. Values are presented as mean ± standard deviation (*n* = 9). Different lowercase letters indicate significant differences among different elevations for the same soil depth, and different capital letters indicate significant differences among different subgroups for different soil depths at the same elevation (*p* < 0.05); F–Shannon, fungal alpha diversity (Shannon index); B–Shannon, bacterial alpha diversity (Shannon index). 0–10, 10–20, and 20–30 cm, indicating the 0–10 cm soil layer, 10–20 cm soil layer, and 20–30 cm soil layer, respectively. H3500 and H4300 denote soil at 3500 and 4,300 m elevation, respectively; M3500–4300 and M4300–3500 denote transplanting soil from 3,500 m elevation to M3500–4300; M4300–3500 denotes transplanting soil from elevation 3,500 m to elevation 4,300 m and transplanting soil from elevation 4,300 m to elevation 3,500 m, respectively.

No significant difference (*p* > 0.05) in bacterial alpha diversity in the 0–10 cm and 10–20 cm soil layers was observed after transplanting high-altitude soils (4,300 m above sea level) to a low-elevation land (3,500 m above sea level, *p* > 0.05), whereas a significant increase in bacterial alpha diversity was observed in the 20–30 cm soil layer (*p* < 0.05, Figure 1). In the fungal community, no significant difference in fungal alpha diversity was observed in the 0–10 cm soil layer (*p* > 0.05), and a significant increase in fungal alpha diversity was found between the 10–20 cm and 20–30 cm soil layers (*p* < 0.05, Figure 1).

No significant difference(*p* > 0.05, Figure 1) in bacterial alpha diversity in the 0–10 cm, 10–20 cm, and 20–30 cm soil layers was observed after transplanting low-elevation soils (3,500 m above sea level) to a high-elevation land (4,300 m above sea level). By contrast, in the fungal community, no significant difference in fungal alpha diversity was found between the 0–10 cm and 10–20 cm soil layers, whereas a significant increase in fungal alpha diversity was found in the 20–30 cm soil layer (*p* < 0.05, Figure 1).

**Figure 2:**
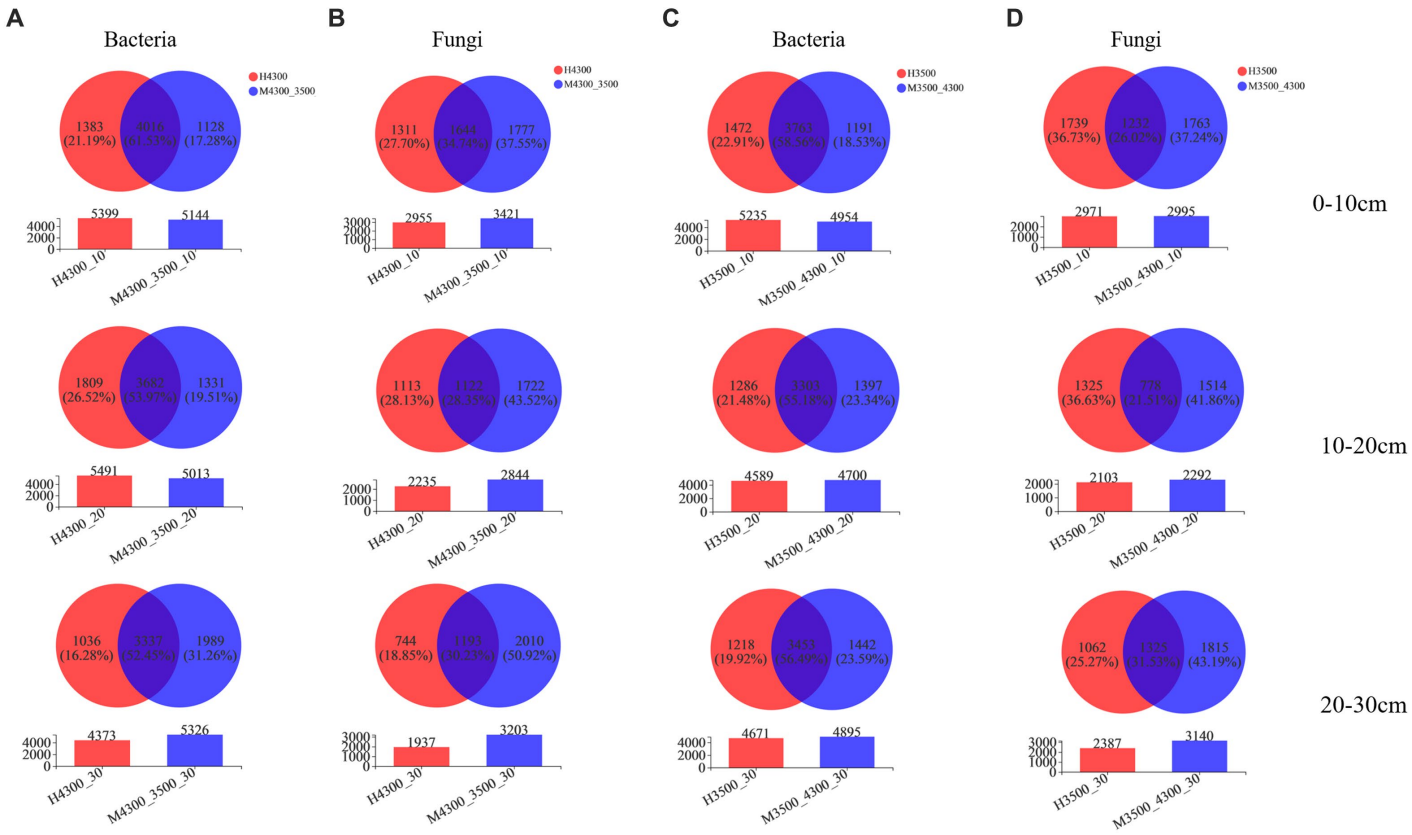
Effect of soil transplantation on soil microbial community species. Changes in bacterial **(A)** and fungal **(B)** OTU counts after transplanting soil from an elevation of 4,300 m to an elevation of 3,500 m. Changes in bacterial **(C)** and fungal **(D)** OTU numbers after transplanting soil from elevation 3,500 m to elevation 4,300 m. 0–10 cm, 10–20 cm, and 20–30 cm denote 0–10 cm soil layer, 10–20 cm soil layer, and 20–30 cm soil layer, respectively. H3500 and H4300 denote elevation 3,500 m and elevation 4,300 m soil, respectively; M3500–4300 and M4300–3500 denote transplanting soil from elevation 3,500 to elevation 4,300 m and transplanting soil from elevation 4,300 to elevation 3,500 m, respectively.

After transplanting soil from a high elevation (4,300 m above sea level) to a low elevation (3,500 m above sea level), the specialization percentage of M3500–4300 was 17.28% in the bacterial community in the 0–10 cm soil layer, 19.51% in the 10–20 cm soil layer, and 31.26% in the 20–30 cm soil layer (Figure 2A). In the fungal community, the specialization percentage of M3500–4300 was 37.55% in the 0–10 cm soil layer, 43.52% in the 10–20 cm soil layer, and 50.92% in the 20–30 cm soil layer (Figure 2B). The specialization percentage of M3500–4300 was higher, which indicated that climate warming had a greater effect on microorganisms in deeper soil layers (Figure 2B).

After transplanting soil from a low elevation (3,500 m above sea level) to a high elevation (4,300 m above sea level), the specialization percentage of M3500-4300 in the bacterial community was 18.53% in the 0–10 cm soil layer, 23.34% in the 10–20 cm soil layer, and 23.59% in the 20–30 cm soil layer (Figure 2C). The specialization rate of M3500–4300 in the fungal community was 37.24% in the 0–10 cm soil layer, 41.86% in the 10–20 cm soil layer, and 43.19% in the 20–30 cm soil layer (Figure 2D). The specialization ratio of fungi was higher than that of bacteria in different soil layers, and the specialization ratio of fungi and bacteria increased with the increase of soil depth.

**Figure 3:**
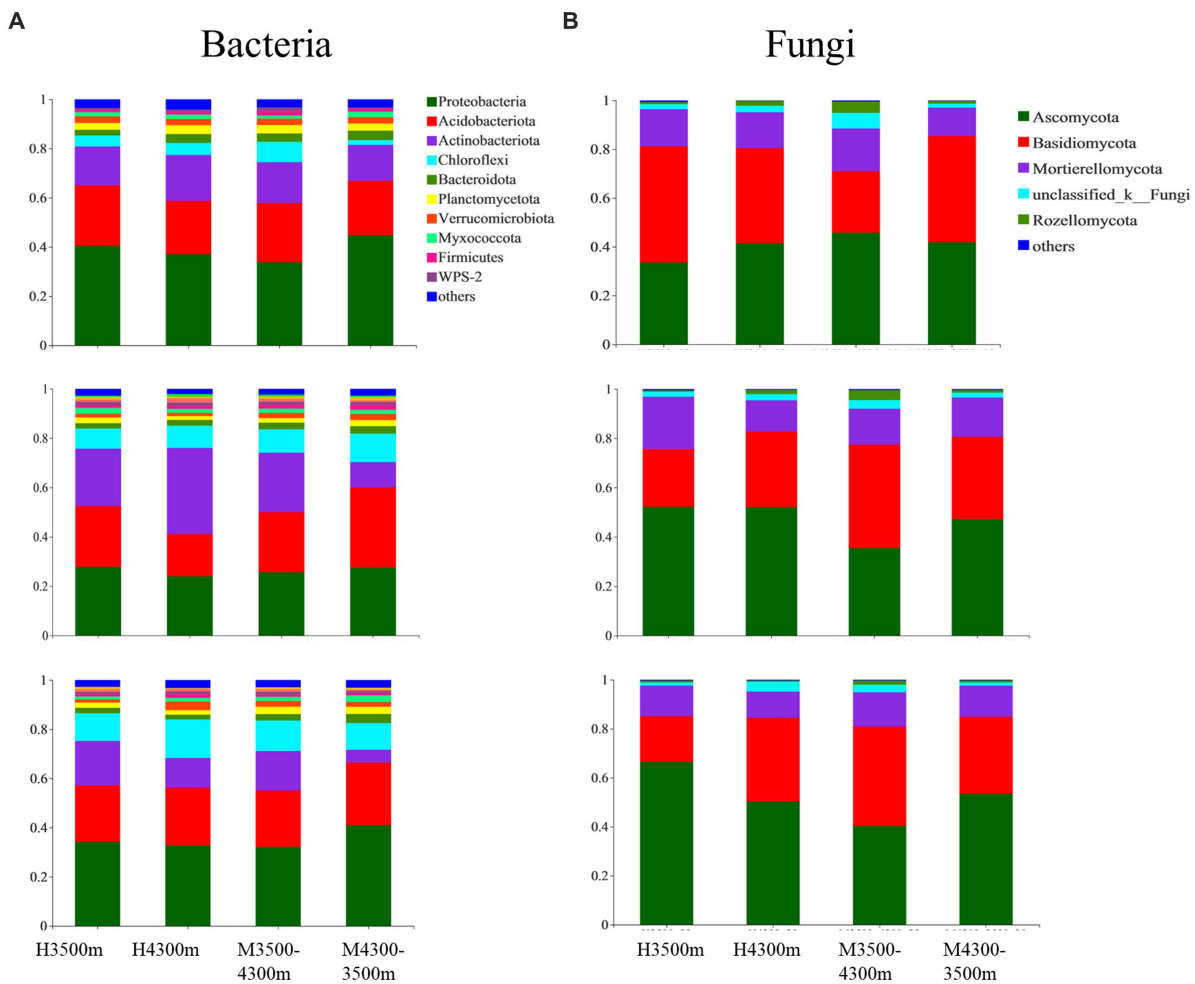
Effect of soil transplantation on **(A)** bacterial and **(B)** fungal communities at the phylum level. H4300 and H3500 denote transplanting soils from 4,300 and 3,500 m above sea level; M4300–3500 denotes transplanting soils from 4,300 to 3,500 m above sea level; H3500 denotes transplanting soils from 3,500 m above sea level; M3500–4300 denotes transplanting soil from elevation 3,500 m to elevation 4,300 m; 0–10 cm, 10–20 cm, and 20–30 cm denote 0–10 cm soil layer, 10–20 cm soil layer, and 20–30 cm soil layer, respectively.

A total of 10 bacterial and five fungal phyla were identified from 108 soil samples with relative abundances above 1%. The average abundance of Proteobacteria (32.89%), Acidobacteriota (24.37%), Chloroflexi (13.61%), and Actinobacteriota (12.84%), as well as the relative abundance of Verrucomicrobiota, Planctomycetota, Bacteroidota, Firmicutes, Myxococcota, WPS-2, and others, were all above 1% and were the dominant phyla in the soil bacterial community (at least the phylum with an average abundance of more than 1% in one sample was considered dominant, Figure 3A). Among soil fungal communities, Basidiomycota had the highest average abundance (45.57%), followed by Ascomycota (34.76%), Mortierellomycota (13.21%), unclassified_k__Fungi (8.56%), and Rozellomycota (1.66%, Figure 3B).

**Figure 4:**
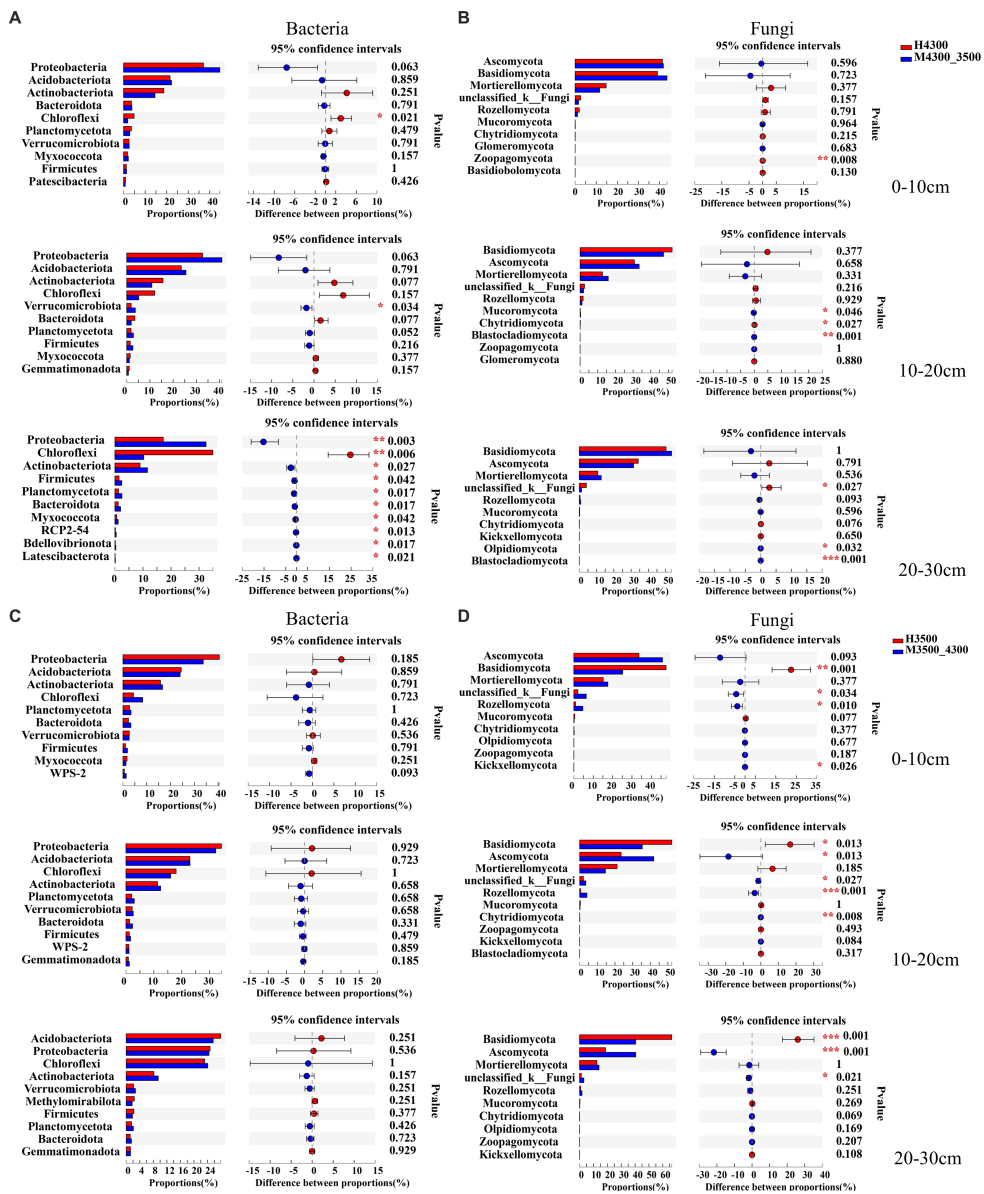
Differences between soil bacterial phylum **(A)** and fungal phylum **(B)** after transplanting soil from 4,300 to 3,500 m above sea level (*n* = 9). Differences in soil bacterial phylum **(C)** and fungal phylum **(D)** after transplanting soil from 3,500 to 4,300 m above sea level (*n* = 9). *p < 0.05; **p < 0.01; ***p < 0.001.

The response of microorganisms to climate warming was different in different soil layers after transplanting soil to 3,500 m at lower elevations. Among the bacterial communities, only Chloroflexi significantly decreased (*p* < 0.05) in the 0–10 cm soil layer, and only Verrucomicrobiota significantly increased (*p* < 0.05) in the 10–20 cm soil layer. In addition, in the 20–30 cm soil layer, the top 10 soil bacterial clades significantly increased in relative abundance (*p* < 0.05), whereas Chloroflexi significantly decreased (*p* < 0.05, Figure 4A). Among fungal communities, only Zoopagomycota showed significant changes (*p* < 0.05) in the 0–10 cm soil layer, and only Mucoromycota, Chytridiomycota, and Blaslocladiomycota showed significant changes (*p* < 0.05) in the 10–20 cm and 20–30 cm soil layers. Moreover, unclassified_k__Fungi, Olpidiomycoa, and Blastocladiomycota had significant changes (*p* < 0.05, Figure 4B). After transplanting the soil to a high elevation of 4,300 m, no significant difference (*p* > 0.05) in the relative abundance of the top 10 bacterial phyla was found in the 0–30 cm soil layer (Figure 4C). In the 0–10 cm soil layer, unclassified_k__Fungi, Basidiomycota, and Rozellomycota increased significantly (*p* < 0.05); in the 10–30 cm soil layer, Basidiomycota decreased significantly (*p* < 0.05), and Ascomycota, unclassified_k__Fungi, and Rozellomycota significantly increased (*p* < 0.05, Figure 4D).

**Figure 5:**
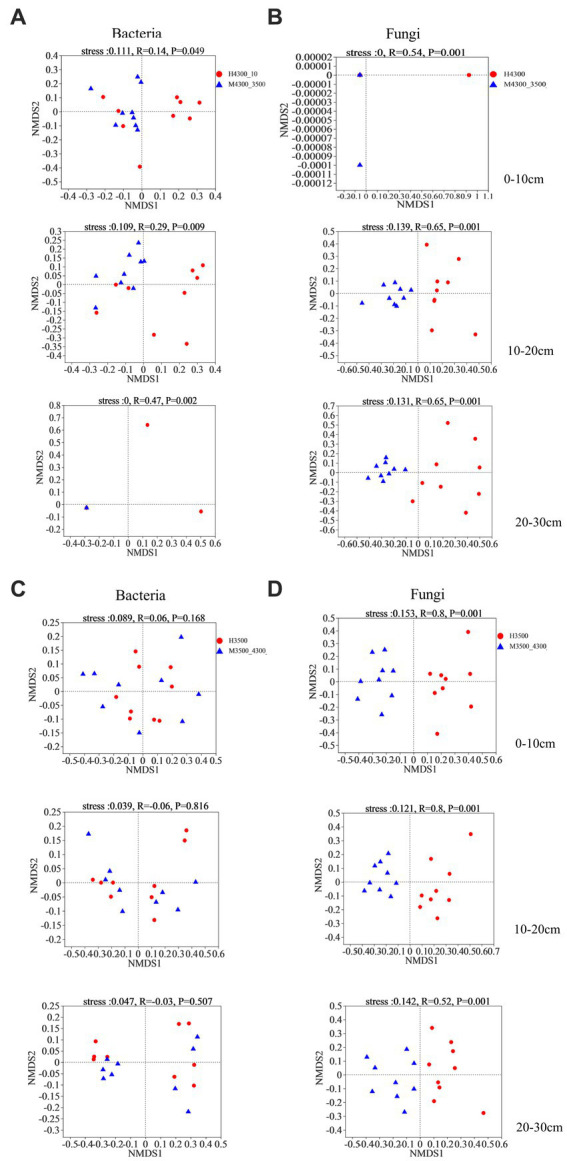
Non–metric multidimensional scaling (NMDS) and similarity analysis (ANOSIM) of the variability of soil bacteria **(A)** and fungi **(B)** based on the Bray–Curtis distance algorithm after transplanting the soil from 4,300 to 3,500 m above sea level. Non–metric multidimensional scaling (NMDS) and similarity analysis (ANOSIM) of soil bacterial **(C)** and fungal **(D)** variability based on the Bray–Curtis distance algorithm after transplanting soil from elevation 3,500 m to elevation 4,300 m. H4300 indicates soil at elevation 4,300 m; M4300–3500 indicates transplanting soil from elevation 4,300 to 3,500 m above sea level; H3500 denotes soils at 3500 and 4,300 m above sea level; M3500–4300 denotes transplanting soils at 3500 m above sea level to 4,300 m above sea level; 0–10 cm, 10–20 cm, and 20–30 cm denote 0–10 cm soil layer, 10–20 cm soil layer, and 20–30 cm soil layer, respectively.

Based on Bray–Curtis differential NMDS and ANOSIM, soil bacteria and fungi were significantly separated in the 0–10 cm, 10–20 cm, and 20–30 cm soil layers after transplanting soil from a high elevation (4,300 m above sea level) to a low elevation (3,500 m above sea level, stress <0.2, *p* < 0.05, Figures 5A,B). After transplanting soil from a low elevation (3,500 m above sea level) to a high elevation (4,300 m above sea level), none of the bacterial communities in the 0–30 cm soil layer were significantly separated (stress <0.2, *p* > 0.05; Figure 5C). By contrast, the fungal communities in the 0–10 cm, 10–20 cm, and 20–30 cm soil layer were separated into two different fungal taxa (stress <0.2, *p* < 0.05; Figure 5D).

### 3.3. Relationship between soil microorganisms and soil physicochemical properties

**Figure 6:**
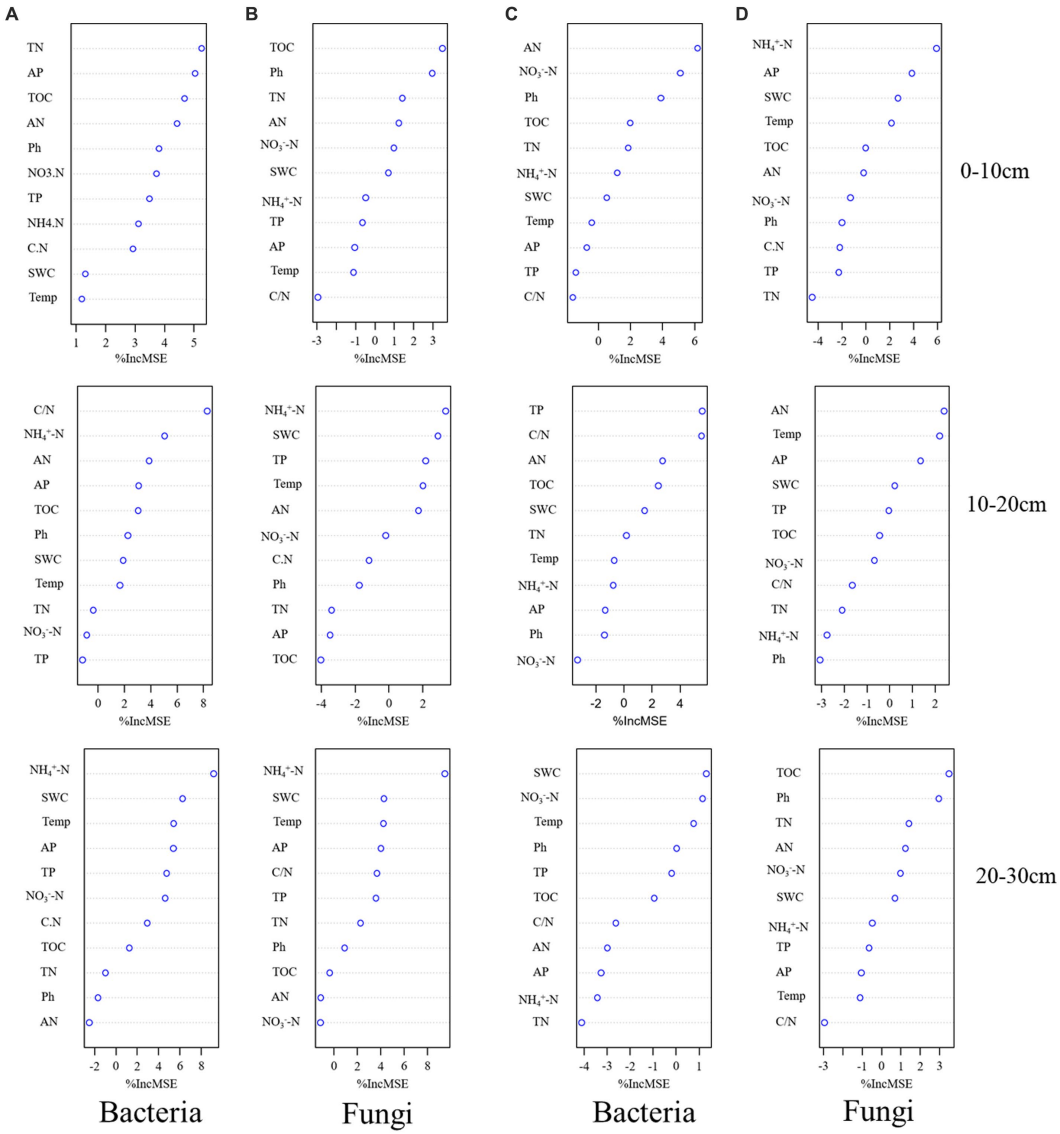
Potential drivers of change in soil bacterial **(A)** and fungal **(B)** alpha diversity after transplanting soils from 4,300 to 3,500 m elevation. Potential drivers of change in soil bacterial **(C)** and fungal **(D)** alpha diversity after transplanting soils from 3,500 to 4,300 m elevation. Soil physicochemical factors were used as RF mean predictive importance (percentage increase in mean square error) of soil microbial alpha diversity. The importance of precision measure was calculated for each tree and averaged over the forest (1,000 trees). The percent increase in mean square error (MSE) of the variables was used to estimate the importance of these predictors, and higher %MSE values indicated more important predictors.

We determined the main microbial predictors of soil microbial alpha diversity after transplanting soil from a high elevation (4,300 m above sea level) to a low elevation (3,500 m above sea level) by RF analysis. In the 0–10 cm soil layer, TN was the best environmental predictor of bacterial alpha diversity (Figure 6A), whereas in the 10–20 cm soil layer, C/N was the best environmental predictor of bacterial alpha diversity (Figure 6A). In the 20–30 cm soil layer, NH_4_^+^-N was the best environmental predictor of bacterial alpha diversity (Figure 6A). In the fungal community, TOC was the best environmental factor to predict fungal alpha diversity in the 0–10 cm soil layer (Figure 6B); NH_4_^+^-N was the best environmental factor to predict bacterial alpha diversity in the 10–20 cm and 20–30 cm soil layers (Figure 6B).

After transplanting soil from a low elevation (3,500 m above sea level) to a high elevation (4,300 m above sea level), AN was the best environmental factor to predict bacterial alpha diversity in the 0–10 cm soil layer (Figure 6C). TP was the best environmental factor to predict bacterial alpha diversity in the 10–20 cm soil layer (Figure 6C), and SWC was the best environmental factor to predict bacterial alpha diversity in the 20–30 cm soil layer (Figure 6C). Among fungal communities, NH_4_^+^-N, AN, and TOC were the best environmental factors to predict fungal alpha diversity of soil layers in 0–10 cm, 10–20 cm, and 20–30 cm soils, respectively (Figure 6D).

### 3.4. Effect of soil transplantation on soil microbial community structure

**Figure 7:**
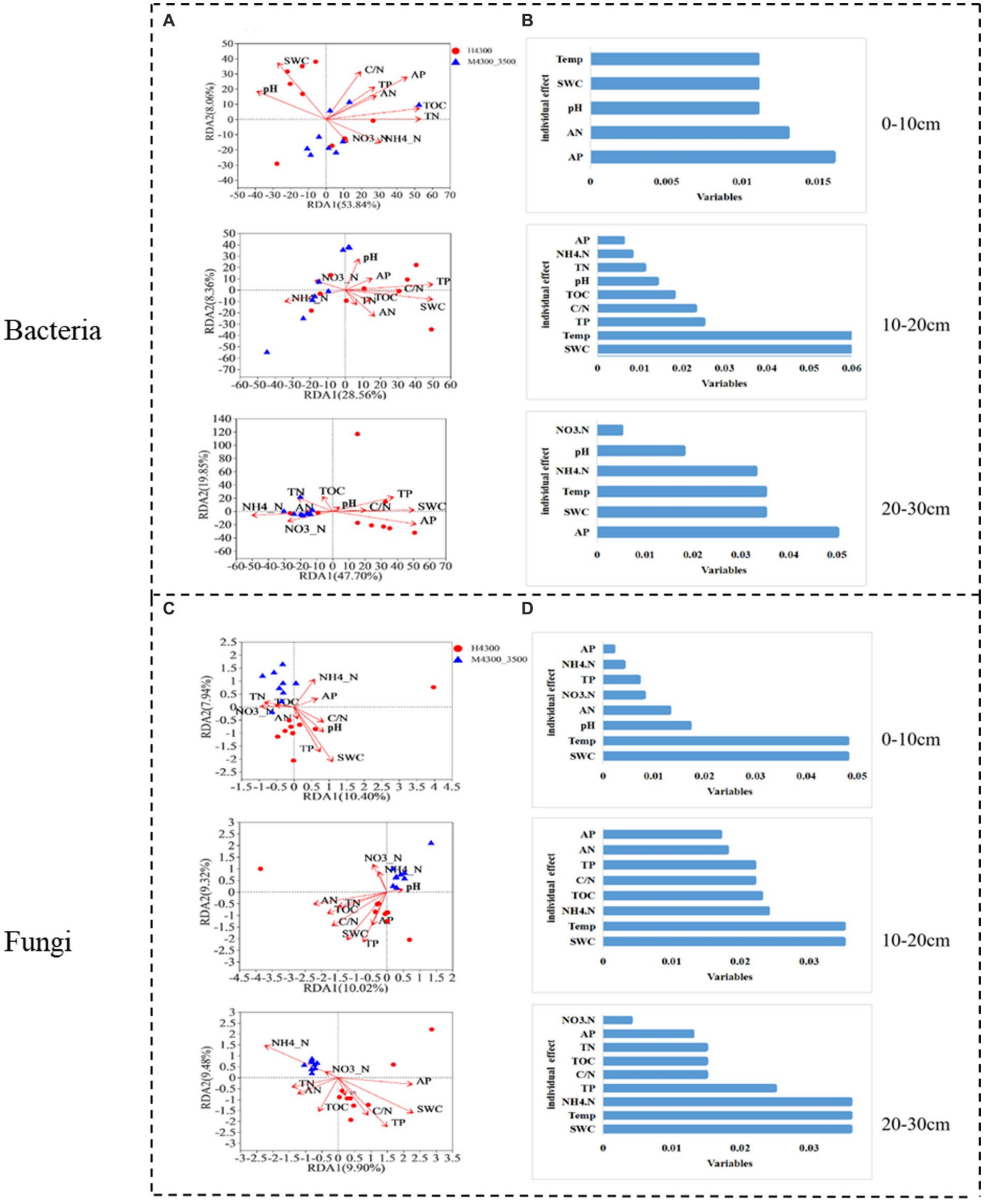
Relationship and hierarchical analysis of microbial community bacteria **(A,B)** and fungi **(C,D)** with soil physicochemical factors after transplanting soil from 4,300 to 3,500 m above sea level. H3500 and H4300, denote soil at 3500 and 4,300 m above sea level, respectively; M4300–3500, denote transplanting soil from 4,300 to 3,500 m; 0–10 cm, 10–20 cm, and 20–30 cm denote 0–10 cm, 10–20 cm, and 20–30 cm soil layers, respectively. NH_4_^+^–N: ammonia nitrogen; NO_3_^−^–N: nitrate nitrogen; AP: available phosphorus; AN: available nitrogen; pH: soil pH; TOC: total organic carbon; TN: total nitrogen; TP: total phosphorus; SWC: soil water content; Soil Temp: soil temperature.

After transplanting soil from a high elevation (4,300 m above sea level) to a low elevation (3,500 m above sea level), the first RDA dimension accounted for 53.84% of the variation in the soil bacterial community in the 0–10 cm soil layer, and the second dimension accounted for 8.06% (61.9% total). TN, TOC, and AP had significant effects (*p* < 0.05) on the bacterial community in the 0–10 cm soil layer (Figure 7A). In the fungal community, the first RDA dimension accounted for 10.4% of the community variation, and the second dimension accounted for 7.94% (18.34% total). TP, SWC, and Temp had a significant effect (*p* < 0.05) on the fungal community (Figure 7C). Hierarchical division analysis showed that AP, SWC, and Temp were the most important factors affecting bacterial and fungal communities, respectively (Figures 7B,D).

In the 10–20 cm soil layer, the first RDA dimension accounted for 38.56% of the variation in the soil bacterial community, and the second dimension accounted for 8.36% (46.29% total). TP, SWC, and Temp had a significant effect on the bacterial community (*p* < 0.05, Figure 7A). In the fungal community, the first RDA dimension accounted for 10.32% of the variation in the soil bacterial community, and the second dimension accounted for 9.02% (19.34% total). In addition, TOC, TP, AN, C/N, SWC, and Temp had a significant effect (*p* < 0.05) on the fungal community (Figure 7C). Hierarchical division analysis showed that SWC and Temp remarkably affected bacterial and fungal communities (Figures 7C,D).

In the 20–30 cm soil layer, the first RDA dimension accounted for 47.7% of the variation in the soil bacterial community, and the second dimension accounted for 9.85% (57.55% total). NH_4_^+^-N, TP, AP, and SWC had a significant effect (*p* < 0.05) on the 20–30 cm bacterial community (Figure 7A). In the soil fungal community, the first RDA dimension accounted for 9.52% of the community variation, and the second dimension accounted for 7.73% (17.35% total). NH_4_^+^-N, TN, TOC, TP, SWC, and Temp had a significant effect (*p* < 0.05) on the bacterial community (Figure 7C). Hierarchical partitioning analysis showed that AP, NH_4_^+^-N, SWC, and Temp were the most important factors affecting bacterial and fungal communities (Figures 7B,D).

**Figure 8:**
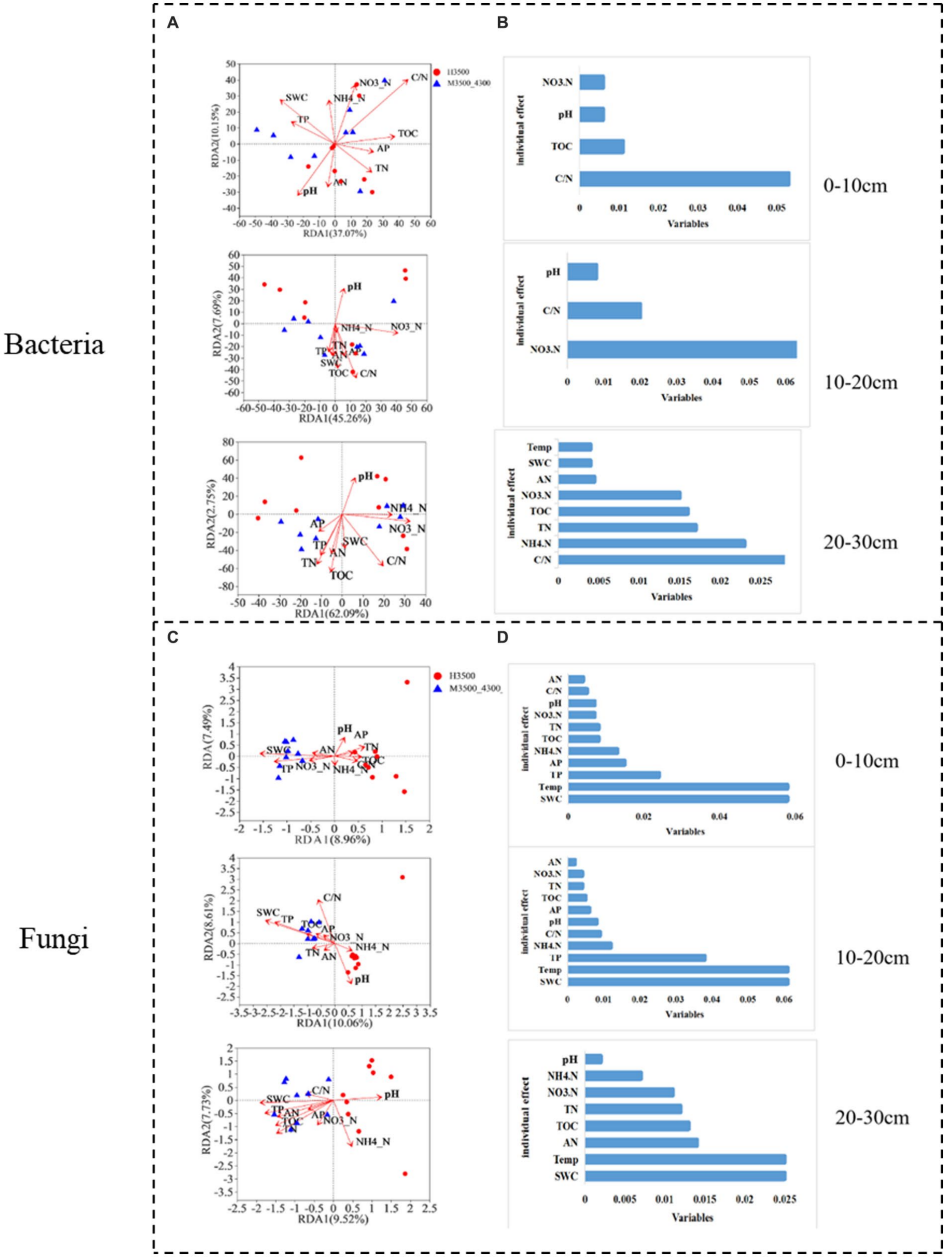
Relationship and hierarchical analysis of microbial community bacteria **(A,B)** and fungi **(C,D)** with soil physicochemical factors after transplanting soil from 3,500 to 4,300 m above sea level. M3500–4300 denotes transplanting soil from 3,500 to 4,300 m above sea level. NH_4_^+^–N: ammonia nitrogen; NO_3_^−^–N: nitrate nitrogen; AP: available phosphorus; AN: available nitrogen; pH: soil pH; TOC: total organic carbon; TN: total nitrogen; TP: total phosphorus; SWC: soil water content; Soil Temp: soil temperature.

After transplanting soil from a low elevation (3,500 m above sea level) to a high elevation (4,300 m above sea level), the first RDA dimension accounted for 37.7% of the soil bacterial community variation, and the second dimension accounted for 10.15% (47.85% total) in the 0–10 cm soil layer. C/N and TOC had a significant effect (*p* < 0.05) on the bacterial community (Figure 8A). In the fungal community, the first RDA dimension accounted for 8.96% of the community variation, and the second dimension accounted for 7.49% (16.45% in total). TP, SWC, and Temp had a significant effect (*p* < 0.05) on the fungal community (Figure 8C). Hierarchical division analysis showed that C/N, SWC, and Temp were the most important factors affecting bacterial and fungal communities (Figures 8B,D).

In the 10–20 cm soil layer, the first RDA dimension accounted for 45.26% of the variation in the soil bacterial community, and the second dimension accounted for 7.69% (53.95% in total). NO_3_^−^-N and C/N had a significant effect (*p* < 0.05) on the 20–30 cm bacterial community (Figure 8A). In the fungal community, the first RDA dimension accounted for 10.06% of the variation in the soil bacterial community, and the second dimension accounted for 8.61% (total 18.67%). TP, pH, C/N, SWC, and Temp had significant effects on the fungal community (*p* < 0.05; Figure 8A). Hierarchical division analysis showed that NO_3_^−^-N, SWC, and Temp were the most important factors affecting bacterial and fungal communities (Figures 8B,D).

In the 20–30 cm soil layer, the first RDA dimension accounted for 62.09% of the variation in the soil bacterial community, and the second dimension accounted for 2.75% (64.84% in total). SWC, Temp, TN, and TOC had significant effects (*p* < 0.05) on the 20–30 cm bacterial community (Figure 8A). In the fungal community, the first RDA dimension accounted for 9.52% of the community variation, and the second dimension accounted for 7.73% (17.25% in total). NH_4_^+^-N, TN, TOC, SWC, and Temp had a significant effect (*p* < 0.05) on the fungal community (Figure 8A). Hierarchical division analysis showed that C/N, SWC, and Temp were the most important factors affecting bacterial and fungal communities (Figures 8B,D).

### 3.5. Effect of soil transplantation on soil microbial co–occurrence networks

**Figure 9:**
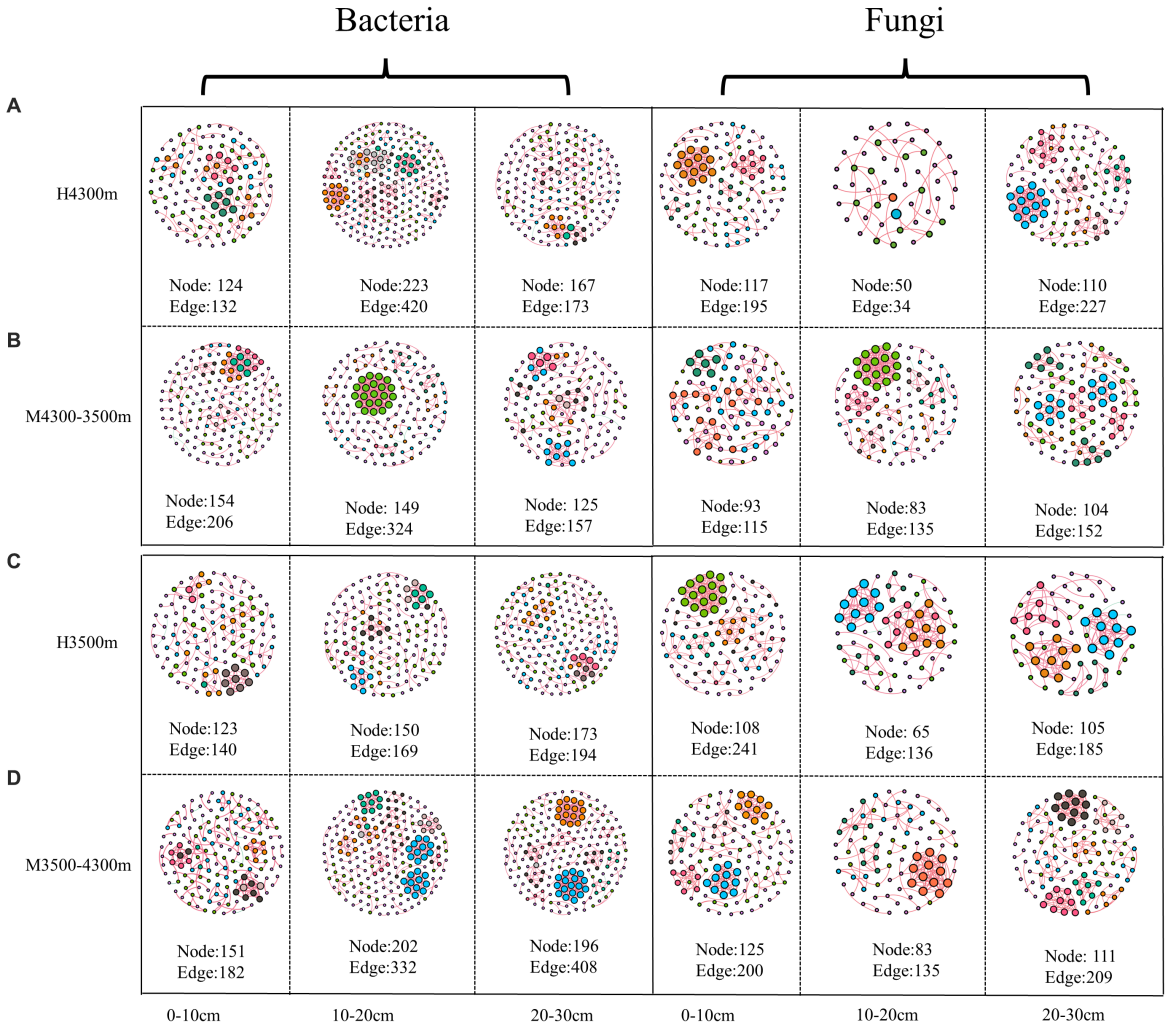
Effect of soil transplantation on soil microbial networks. **(A)** Co–occurrence network of soil bacteria and fungi *in situ* at 4300 m elevation; **(B)** co–occurrence network of soil bacteria and fungi after transplanting soil from 4,300 to 3,500 m elevation; **(C)** co–occurrence network of soil bacteria and fungi *in situ* at 3,500 m elevation; **(D)** co–occurrence network of soil bacteria and fungi after transplanting soil from 3,500 to 4,300 m elevation. Each node represents a bacterial and fungal taxonomic unit at the genus level, and the node size is proportional to the relative abundance of all OUTs. The size of each node is proportional to the relative abundance of the corresponding taxonomic unit, and network nodes with the same degree are randomly assigned to the same color. Red indicates a positive correlation, and blue indicates a negative correlation. Edges indicate a significant correlation between two corresponding taxa (*r* > 0.6, *p* < 0.01). The thickness of each edge is proportional to the corresponding correlation coefficient. Microbial Alpha Diversity.

In estimating the complexity of the network, the number of nodes and edges, the ratio of positive and negative edges, clustering coefficient, and average degree were considered. The number of nodes and edges were used to determine the size of the network, whereas the ratio of positive and negative edges provided insight into the balance of the network. The clustering coefficient was used to measure the degree of node clustering, and the average degree was used to determine the average number of edges per node. After transplanting soil from a high elevation (4,300 m above sea level) to a low elevation (3,500 m above sea level), the proportion of negative connections and the total number of edges in the network nodes increased in the 0–10 cm soil layer bacterial community, whereas the average clustering coefficient decreased, indicating that climate warming increased the complexity of the bacterial community network (Figure 9A; Table 3). In the 10–20 cm and 20–30 cm soil layers, the network nodes, total number of edges, average degree, and average clustering coefficient decreased. Therefore, climate warming reduced the complexity of the 10–30 cm soil bacterial network (Figure 9A; Table 3). In the fungal community, fungal network nodes, mean clustering coefficient, total edge number, and mean degree in the 0–10 cm and 20–30 cm soil layers decreased after climate warming, indicating that climate warming reduced the complexity of the fungal network (Figure 9B; Table 3). In the 10–20 cm soil layer, the mean degree of the fungal network, mean clustering coefficient, number of network nodes, and total edges increased, indicating that climate warming increased the complexity of the fungal network in the 10–20 cm soil layer (Figure 9B; Table 3).

**Table 3:** Effect of soil transplantation on the topological properties of soil microorganisms.

Soil depth	Topological properties	4,300 m	M4300–3500 m	3,500 m	M3500–4,300 m
	Bacteria				
0–10 cm	Number of nodes	124	154	123	151
	Number of edges	132	206	140	182
	Average degree	2.129	2.675	2.27	2.41
	Modularity	0.953	0.801	0.92	0.96
	Average clustering coefficient	0.769	0.863	0.79	0.83
	Average path length	1.238	1.5	1.466	1.222
10–20 cm	Number of nodes	223	149	150	202
	Number of edges	420	324	169	332
	Average degree	4.249	3.767	2.253	3.287
	Modularity	0.876	0.574	0.93	0.97
	Average clustering coefficient	0.939	0.776	0.777	0.859
	Average path length	2.295	1.073	1.464	1.229
20–30 cm	Number of nodes	167	125	173	196
	Number of edges	173	157	194	408
	Average degree	2.072	2.512	2.243	4.163
	Modularity	0.914	0.88	0.81	0.93
	Average clustering coefficient	0.864	0.726	0.827	0.883
	Average path length	1.52	1.165	1.332	0.121
Soil depth	Fungi				
0–10 cm	Number of nodes	117	93	108	125
	Number of edges	195	115	241	200
	Average degree	3.333	2.473	4.463	3.2
	Modularity	0.939	0.808	0.725	0.855
	Average clustering coefficient	0.932	0.925	0.985	0.962
	Average path length	1.035	1.101	1.044	1.02
10–20 cm	Number of nodes	50	83	65	83
	Number of edges	34	135	136	135
	Average degree	1.36	3.253	4.185	3.253
	Modularity	0.741	0.922	0.922	0.741
	Average clustering coefficient	0.844	0.979	0.844	0.979
	Average path length	1.171	1.004	1.007	1.007
20–30 cm	Number of nodes	110	104	105	111
	Number of edges	227	152	185	209
	Average degree	4.127	2.923	3.524	3.766
	Modularity	0.924	0.804	0.85	0.87
	Average clustering coefficient	1	0.973	0.974	0.963
	Average path length	1	1	1.016	1.064

After transplanting soil from a low elevation (3,500 m above sea level) to a high elevation (4,300 m above sea level), in the bacterial community, the proportion of connections, mean degree, mean clustering coefficient, network nodes, and total number of edges in the 0–10 cm, 10–20 cm, and 20–30 cm soil layers increased, indicating that the complexity of the soil bacterial network increased after climate warming (Figure 9C; Table 3). In the fungal community, the proportion of fungal network nodes and negative connections in the 0–10 cm soil layer increased after climate cooling, but the total network edge, mean degree, and mean clustering coefficient decreased after climate cooling (Figure 9B; Table 3). Network nodes, mean clustering coefficient, and proportion of negative connections in the 0–10 cm and 10–20 cm soil layers increased after climate cooling; the mean degree decreased, and the total network edge did not change (Figure 9C; Table 3). The network nodes, total network edges, mean degree, and proportion of negative connections in the 30 cm soil layer increased, but the mean clustering coefficient decreased (Figure 9D; Table 3).

## 4. Discussion

### 4.1. Effect of soil transplantation on soil microbial alpha diversity

We hypothesized that soil transplantation would change the soil bacterial and fungal alpha diversity (Shannon index); however, this study found no significant change in fungal and bacterial Shannon indices in the 0–10 cm soil layer either by transplanting the soil to a warmer or colder location (Figure 1), whereas the fungal Shannon index in the 10–20 cm and 20–30 cm soil layers as well as the bacterial Shannon index in the 20–30 cm soil layer increased significantly after transplanting the soil to a warmer location (Figure 1). In addition, only the fungal Shannon index in the 20–30 cm soil layer increased significantly after transplanting the soil to a colder location (Figure 1). This finding is different from our original hypothesis. In general, temperature is an important factor affecting the activity and efficiency of organisms in the soil and controlling soil nutrient mineralization, apoplastic decomposition, and soil organic carbon stability (Pries et al., 2012; Palareti et al., 2016). Temperature changes can lead to changes in the diversity and composition of soil microbial communities. This result is different from that of previous studies (Looby et al., 2016; Cheng et al., 2017; Nottingham et al., 2018) probably because reduced precipitation neutralizes the positive effects of soil warming on soil microbial communities in *A. georgei* var. *smithii* forests (Liu et al., 2022). After transplanting soil to a warmer location, RF analysis showed that TN and TOC were the best environmental factors predicting the alpha diversity (Shannon index) of bacteria and fungi in the 0–10 cm soil layer (Figures 6A,B), respectively. Related studies have shown that TOC and TN in soil provide carbon sources and nutrients for soil microorganisms (Pan et al., 2021), and changes in their levels will cause changes in soil microbial diversity. However, this study found no significant changes in soil TOC and TN after transplanting the soil to a warmer location (Table 2), which causes the lack of significant differences in the alpha diversity (Shannon index) of fungi and bacteria in the 0–10 cm soil layer. C/N and NH_4_^+^-N were the best environmental factors predicting changes in fungal and bacterial diversity in the 10–20 cm and 20–30 cm soil layers after climate warming (Figures 6A,B). Soil nutrients such as N and P are essential nutrients for microbial growth, and changes in their content and effectiveness affect microbial composition and diversity (Liu et al., 2018; Pan et al., 2021). Soil C/N ratio can reflect the quality of the substrate for soil microbial growth, and it is usually considered as a sensitive indicator of soil quality (Wan et al., 2015), which can measure soil C and N nutrient balance. Moreover, a low C/N ratio can accelerate microbial decomposition and the rate of N mineralization. Based on RF analysis, N and P in soil were identified as the most influential environmental factors predicting changes in fungal and bacterial diversity in the 0–10 cm and 10–20 cm soil layers after climate cooling (Figures 6C,D). As previously mentioned, alterations in the availability of N and P, which are essential nutrients for microbial growth, and P, which is required for rapid growth during ribosome, ATP, DNA, and RNA production, can affect microbial composition and diversity (Delgado-Baquerizo et al., 2017). In this study, we found no significant differences in TN, TP, AN, and AP contents in the 0–10 cm and 10–20 cm soil layers after transplanting the soil to colder locations (Table 2). SWC and TOC were the best predictors of changes in bacterial and fungal diversity in the 20–30 cm soil layer, respectively. Soil nutrient content decreases with the increase of soil layers (Wang et al., 2016; Song et al., 2022), resulting in a limitation of nutrients required by fungi and bacteria.

### 4.2. Effect of soil transplantation on soil microbial community structure and composition

In this study, the structure of all soil fungal and bacterial communities changed after transplanting the soil to a warmer location (Figures 5A,B), which verifies our hypothesis and indicates that this ecosystem has higher sensitivity to a warmer climate (DeAngelis et al., 2015; Rui et al., 2015; Treseder et al., 2016; Cheng et al., 2017; Yu et al., 2019). By contrast, only the fungal community structure was altered after transplanting the soil to a colder location (Figures 5C,D), which is different from our prediction. Soil temperature and water content were the main drivers of changes in fungal community structure, whereas bacterial community structure was related to changes in soil environmental conditions, indicating that fungi are more sensitive to climate changes than bacteria. This finding is consistent with the results of Sun et al. (2022). Compared with bacteria, fungi have better resistance to low-temperature stress and water stress (DeAngelis et al., 2015; Palareti et al., 2016). Soil fungi have a unique mycelial structure, and they likely grow toward water resources than prokaryotes (Torsvik and Øvreås, 2008). Furthermore, high-elevation areas of the Sygera Mountains have abundant precipitation, and soil pores filled with water can interconnect, which promotes spore/mycelial diffusion in the soil. This increase in the randomness of fungal community composition under the same vegetation type leads to significant differences in community composition.

The relative abundance of the dominant bacterial phylum in the 0–10 cm and 10–20 cm soil layers and the dominant fungal phylum in all soil layers did not change significantly after transplanting the soil to a warmer location (Figures 4A,B), whereas the dominant bacterial phylum in the 20–30 cm soil layer maintained a higher activity after warming (Figures 4A,B). Therefore, fungi and bacteria adapt differently to warming in different soil layers possibly because low precipitation neutralizes the positive effects of surface soil warming on the soil microbial community (Liu et al., 2022). No significant changes in the dominant bacterial phylum were observed in all soil layers after transplanting the soil to colder sites (Figures 4C,D), whereas fungi maintained better activity. Similar results were found by Liang et al. (2015) and Toju et al. (2017). Apoplastic litter is usually stubborn at high elevations because of nutrient limitation, leaf thickness, and increased sclerosis (Schneider et al., 2012), a pattern that favors the presence of fungi, which can break down organic matter that is more recalcitrant than many bacteria (Floudas et al., 2012; Schneider et al., 2012). In addition, fungal activity appears to be less affected by soil shifts than bacteria because fungi are generally more tolerant to water stress than prokaryotes (Building, 2009; Zumsteg et al., 2013) and their mycelial structures. Fungi also readily grow toward water resources than prokaryotes (Torsvik and Øvreås, 2008). Therefore, fungi have a growth advantage at lower temperatures.

### 4.3. Effect of soil transplantation on soil microbial community networks

We hypothesized that experimental climate warming would increase the complexity of species associations and vice versa. Previous studies have shown that network complexity is positively correlated with ecosystem function (Wagg et al., 2019), and an increase in network complexity can improve the efficiency of ecosystem energy flow and material cycling, which in turn has a positive effect on ecosystem function (Högberg et al., 2007). The present study found that transplanting soil to warmer locations increased the complexity of bacterial community networks in the 0–10 cm soil layer (Figures 9A,B; Supplementary Table 3), which is similar to the results of Yuan et al. (2021). However, the complexity of the bacterial network in the 10–20 cm and 20–30 cm soil layers decreased (Figures 9A,B; Supplementary Table 3), which was different from our original hypothesis. This result is due to the associated environmental changes (reduced moisture and soil nutrients) caused by increased soil depth, which becomes a strong filtering factor for microbial species (Stegen et al., 2012; Wang et al., 2016; Song et al., 2022), resulting in reduced bacterial network complexity in deeper soil layers. In the fungal community, fungal network complexity in the 0–10 cm and 20–30 cm soil layers decreased after climate warming (Figures 9A,B; Supplementary Table 3). By contrast, in the 10–20 cm soil layer, fungal network complexity increased after climate warming (Figures 9A,B; Supplementary Table 3). Fungi are more adapted to soil environments with lower temperatures and higher water content (DeAngelis et al., 2015; Palareti et al., 2016), which allows high-elevation soils to have more fungal diversity and more connected relationships, thereby building more complex networks. After warming, fungi are not only subjected to adaptive stress but also limited by soil nutrients and light. Thus, in the face of climate change, fungi will not only adapt to their environment and habitat, but also exhibit more structural complexity loss, resulting in a reduction in fungal network complexity. By contrast, the complexity of the 10–20 cm fungal network increased probably because the environmental conditions in this layer of soil were more suitable for the growth of certain fungi, leading to an increase in fungal network complexity.

The complexity of the bacterial network in the 0–10 cm, 10–20 cm, and 20–30 cm soil layer increased after transplanting the soil to a colder location (Figures 9C,D; Supplementary Table 3) because the increase in SWC improved the ability of bacteria to resist low-temperature stress (Sang et al., 2021). On the contrary, in the fungal community, the nodes of the fungal network increased in the 0–10 cm and 10–20 cm soil layers (Figures 9C,D; Supplementary Table 3), but the total edge of the network decreased probably because lower temperatures break the mutualistic relationship among soil fungal communities.

Ecological networks with a high proportion of negative and positive correlations among microbial taxa are adaptable to environmental changes (Coyte et al., 2015; Hernandez et al., 2021), with positive correlations representing positive interactions between taxa and negative correlations representing negative interactions. However, a highly relative proportion of positive correlations can affect community stability because when a microbial community is disturbed, that is, the number of one member of a positive feedback loop decreases, the fitness of other taxa that depend on the feedback loop services will be negatively affected (Stouffer and Bascompte, 2011; Suweis et al., 2014; Coyte et al., 2015). In addition, negative interactions will reduce the co-oscillation of microsomal communities (Coyte et al., 2015; Zhong et al., 2022). In this study, the positive and negative correlation ratios in the microbial networks of different soil layers were changed when the soils were transplanted to warmer or cooler places (Supplementary Table 3), but the positive correlation ratios remained predominant, which indicates that the soil microbial community in this study area is unstable, and it responds strongly to climate change.

## 5. Conclusion

The results of this study showed that the microbial composition and community structure of different soil layers responded differently to climate change (warming and cooling). Soil transplantation (simulated climate warming and cooling) had no significant effect on soil microbial diversity in the surface layer (0–10 cm) and a greater effect on microbial diversity in the 10–20 and 20–30 cm soil layers. Warming altered the structure of microbial communities in all soil layers, whereas cooling had no significant effect on the structure of bacterial communities in all soil layers (0–10 cm, 10–20 cm, and 20–30 cm) but had a significant effect on fungal communities in all soil layers. Soil moisture content and temperature were the main drivers of the differences in soil fungal community structure, whereas soil bacterial community structure was more closely related to soil conditions. Climatic cooling increased the complexity of bacterial and fungal networks in all soil layers and the 20–30 cm soil layer. For the 0–20 cm soil fungal network, climatic cooling only increased the number of network nodes and decreased the total number of network edges. In addition, in this study, climate change did not change the symbiotic pattern of soil microorganisms, and the symbiotic relationship among soil microbial communities was dominant. The changes in microbial community structure induced by soil transplantation are important for predicting the response of alpine forest ecosystems to global changes (warming and cooling). Moreover, studies of microbial community dynamics provide new insights into predicting future carbon stability.

## Data availability statement

The datasets presented in this study can be found in online repositories. The name of the repository and accession number can be found at: NCBI; PRJNA955721.

## Author contributions

FF conceived the concept for this study and designed the methodology, collected the data, analyzed the data, and wrote the manuscript. JL conceived the concept for this study and designed the methodology, and collected the data. WC, HD, SX, and YL collected the data. All authors contributed to the article and approved the submitted version.

## Funding

This work was funded by the National Ministry of Science and Technology Ecological Station (LZF2020–2025), the Longterm Ecological Observation Study of Alpine Pine in Southeast Tibet (Science and Technology Innovation Base) (XZ202301JD0001G), and The Independent Research Project of Science and Technology Innovation Base in Tibet Autonomous (XZ2022JR0007G).

## Conflict of interest

The authors declare that the research was conducted in the absence of any commercial or financial relationships that could be construed as a potential conflict of interest.

## Publisher’s note

All claims expressed in this article are solely those of the authors and do not necessarily represent those of their affiliated organizations, or those of the publisher, the editors and the reviewers. Any product that may be evaluated in this article, or claim that may be made by its manufacturer, is not guaranteed or endorsed by the publisher.
