# Supplementary material for: Simulating the effect of climate change on soil microbial community in an Abies georgei var. smithii forest
Source: Front Microbiol. 2023 Jun 2;14:1189859. doi: 10.3389/fmicb.2023.1189859 (PMC10272780; doi:10.3389/fmicb.2023.1189859)
Supplement: Supplementary file 1 [file Table_1.DOCX]

Supplementary Material

**Simulating the effect of climate change on soil microbial community in an *Abies georgei* var. *smithii* forest**

**Fangwei Fu***, **Yueyao Li** , **Wensheng Chen**, **Huihui Ding**, **Siying Xiao**

*** Correspondence:** Jiangrong Li:[ljrong06@xza.edu.cn](mailto:ljrong06@xza.edu.cn) (J. Li)

# Supplementary Figures and Tables

Table 3. Effect of soil transplantation on the topological properties of soil microorganisms.

| Soil Depth | Topological properties | 4300m | M4300-3500m | 3500m | M3500-4300m |
| --- | --- | --- | --- | --- | --- |
|  | Bacteria |  |  |  |  |
| 0-10cm | Number of nodes | 124 | 154 | 123 | 151 |
|  | Number of edges | 132 | 206 | 140 | 182 |
|  | Aversge degree | 2.129 | 2.675 | 2.27 | 2.41 |
|  | Modularity | 0.953 | 0.801 | 0.92 | 0.96 |
|  | Aversge clustering coefficient | 0.769 | 0.863 | 0.79 | 0.83 |
|  | Aversge path length | 1.238 | 1.5 | 1.466 | 1.222 |
| 10-20cm | Number of nodes | 223 | 149 | 150 | 202 |
|  | Number of edges | 420 | 324 | 169 | 332 |
|  | Aversge degree | 4.249 | 3.767 | 2.253 | 3.287 |
|  | Modularity | 0.876 | 0.574 | 0.93 | 0.97 |
|  | Aversge clustering coefficient | 0.939 | 0.776 | 0.777 | 0.859 |
|  | Aversge path length | 2.295 | 1.073 | 1.464 | 1.229 |
| 20-30cm | Number of nodes | 167 | 125 | 173 | 196 |
|  | Number of edges | 173 | 157 | 194 | 408 |
|  | Aversge degree | 2.072 | 2.512 | 2.243 | 4.163 |
|  | Modularity | 0.914 | 0.88 | 0.81 | 0.93 |
|  | Aversge clustering coefficient | 0.864 | 0.726 | 0.827 | 0.883 |
|  | Aversge path length | 1.52 | 1.165 | 1.332 | 0.121 |
| Soil Depth | Fungi |  |  |  |  |
| 0-10cm | Number of nodes | 117 | 93 | 108 | 125 |
|  | Number of edges | 195 | 115 | 241 | 200 |
|  | Aversge degree | 3.333 | 2.473 | 4.463 | 3.2 |
|  | Modularity | 0.939 | 0.808 | 0.725 | 0.855 |
|  | Aversge clustering coefficient | 0.932 | 0.925 | 0.985 | 0.962 |
|  | Aversge path length | 1.035 | 1.101 | 1.044 | 1.02 |
| 10-20cm | Number of nodes | 50 | 83 | 65 | 83 |
|  | Number of edges | 34 | 135 | 136 | 135 |
|  | Aversge degree | 1.36 | 3.253 | 4.185 | 3.253 |
|  | Modularity | 0.741 | 0.922 | 0.922 | 0.741 |
|  | Aversge clustering coefficient | 0.844 | 0.979 | 0.844 | 0.979 |
|  | Aversge path length | 1.171 | 1.004 | 1.007 | 1.007 |
| 20-30cm | Number of nodes | 110 | 104 | 105 | 111 |
|  | Number of edges | 227 | 152 | 185 | 209 |
|  | Aversge degree | 4.127 | 2.923 | 3.524 | 3.766 |
|  | Modularity | 0.924 | 0.804 | 0.85 | 0.87 |
|  | Aversge clustering coefficient | 1 | 0.973 | 0.974 | 0.963 |
|  | Aversge path length | 1 | 1 | 1.016 | 1.064 |
